# Oligodendrocyte progenitor cell responses to inflammatory demyelination with aging

**DOI:** 10.21203/rs.3.rs-7614774/v1

**Published:** 2025-09-23

**Authors:** Emily E. Fresenko, Camilla N. Bahri, Noor F. Ahmed, Davin Packer, Benjamin J. Tabor, Wenjing Sun, Michelle A. Wedemeyer, Cole A. Harrington

**Affiliations:** The Ohio State University Wexner Medical Center; The Ohio State University Wexner Medical Center; The Ohio State University Wexner Medical Center; The Ohio State University Wexner Medical Center; The Ohio State University Wexner Medical Center; The Ohio State University; The Ohio State University Wexner Medical Center; The Ohio State University Wexner Medical Center

**Keywords:** aging, adoptive transfer, immune oligodendrocyte, oligodendrocyte, oligodendrocyte progenitor cell, multiple sclerosis, remyelination

## Abstract

Oligodendrocyte progenitor cells (OPCs) have the capacity to self-renew, differentiate into mature myelinating cells, and remyelinate the central nervous system in response to demyelination. Normal aging is associated with a reduction in the functional capacity of OPCs and induces distinct transcriptional signatures even in the absence of an autoimmune inflammatory demyelination insult. To determine how aging impacts the OPC response to an acute inflammatory insult comparable to a demyelinating event in multiple sclerosis (MS), we performed adoptive transfer of young myelin-reactive Th17 T cells into young and aged mice. Spinal cord OPC responses were quantified using lineage tracing and myelin sheath thickness was quantified using transmission electron microscopy. In the subacute phase 9–10 days after adoptive transfer, the density of both young and aged OPCs is enriched in spinal cord lesions compared to non-lesion white matter. After adoptive transfer, the density of aged OPCs is significantly higher than naïve/non-adoptive transfer aged spinal cord. Differentiated oligodendrocytes (OLs) are relatively preserved within lesions of aged and young animals despite an overall reduction in OL density after adoptive transfer. While lineage tracing identified newly formed oligodendrocytes after adoptive transfer in both young and aged lesions, less oligodendrocyte differentiation was observed in aged animals. Despite the reduction of OPC differentiation in aged animals, there was no significant difference in the extent of remyelination observed for young and aged lesions. Aged OPCs rise to the challenge in response to a strong auto-immune attack, suggesting that compensatory strategies allow both young and aged OPCs to survive and remyelinate in the inflammatory environment. Identifying pathways that promote resilience of young and aged OPCs in the face of an inflammatory challenge will facilitate the development of remyelinating therapies for the treatment of people with MS across the full spectrum of human aging.

## Background

In the postnatal central nervous system (CNS), oligodendrocyte progenitor cells (OPCs) are lineage restricted progenitors with the capacity to proliferate, migrate, and differentiate into mature myelinating oligodendrocytes during development and adulthood (1–3) and in response to demyelinating injury (4, 5). Multiple sclerosis (MS) is a chronic demyelinating disorder of the CNS in which an autoimmune attack of oligodendroglia and their myelin processes results in demyelinating lesions (6–8). OPCs and maturing oligodendrocytes have been identified in MS lesions from patients of all ages (9, 10) suggesting that OPCs may retain the capacity to differentiate and remyelinate after a demyelinating attack despite an inhibitory inflammatory environment (8, 11).

Individuals with MS typically live with the disease for decades and aging is a risk factor for progression and accumulation of neurological disability (12, 13). Cellular aging in MS leads to senescence of immune cells and other CNS resident cell populations (13) which may influence the ability of aged OPC to differentiate and remyelinate. Physiological aging of OPCs is associated with phenotypic changes in ion channel expression (14), reduced proliferation and differentiation (14, 15) and increased cellular senescence with upregulation of immune response pathways (14–16). Developmental inhibitors of OPC differentiation, e.g. Wnt and hypoxia inducible factor-1 alpha (HIF-1a) pathways, are upregulated in aged OPCs and pharmacological inhibition of these pathways promotes differentiation of aged OPCs *in vitro* (16). Aged OPCs have reduced proliferation, slower recruitment into lesions, and slower differentiation in response to *in vivo* toxin-mediated demyelination (17–19). Metformin plus alternative day fasting (15) and young macrophages (20) can partially rescue the slower remyelination kinetics observed after toxin-mediated demyelination in aged animals. These studies indicate that manipulation of aged OPCs in animal models can boost OPC functionality indicating that remyelination is a potential therapeutic strategy for aged individuals with MS with intact axons.

Formal investigations of the impact of aging on OPCs performance after an auto-immune inflammatory insult have not been conducted to date. While toxin-mediated models benefit from stereotyped OPC responses; the lack of an adaptive immune response does not accurately reflect the microenvironment of human MS lesions. The muted immune response seen with toxin-based models compared to autoimmune demyelinating models may limit the translation of findings into therapies that successfully promote remyelination in people with multiple sclerosis. Furthermore, the use of models without an adaptive autoimmune component may also overlook promising mechanisms that are a direct of result of the autoimmune attack. Transcriptional profiling of MS lesions has consistently revealed a disease-associated transcriptional signature in oligodendrocytes (21, 22) that has been replicated in both inflammatory (22–24) and toxin-based murine models of demyelination (25–27). More detailed transcriptional profiling is warranted to delineate the relationship between the heterogeneous oligodendrocyte subtypes found in animal models of MS and oligodendrocyte populations in human MS tissue will be critical to understanding the extent to which animal models replicate the OPC heterogeneity seen in human disease.

The interpretation of findings from murine inflammatory models is challenging due to the temporal and regional heterogeneity of observed lesions, early axonal degeneration, high inflammatory infiltrate, and high severity of acute disease (28–30). Inflammatory models offer the benefit of a T cell mediated auto-immune process which may simulate acute demyelination in MS (6, 31) and may more accurately reflect oligodendroglial behavior in response to an inflammatory demyelinating event. In addition, disease-associated oligodendroglia may interact with T cells through expression of major histocompatibility complex (MHC) class I and II molecules (23, 32, 33). While disease-associated oligodendroglia are present in toxin-mediated models (25–27, 34) the lack of an autoimmune process may limit our understanding of the behavior of disease-associated oligodendroglia when confronted with a robust inflammatory response such as is seen with MS lesions.

To assess differences in survival, localization, and differentiation of young versus aged OPCs to an autoimmune demyelinating attack we crossed a tamoxifen-inducible OPC Cre line (*Pdgfra*-*Cre*^*ERT*^) (35) with an inducible green fluorescent protein (GFP) reporter line (*RCE*− *Rosa26*^*flstopflEGFP*^) (36) to perform lineage tracing of OPCs after adoptive transfer of young myelin-reactive Th17 T cells. The use of young T cells for adoptive transfer studies prevents confounding of results due to immunosenescence of aged T cells, as observed differences may be attributed solely to differences between OPCs in young and aged animals. Following adoptive transfer, OPCs from both young and aged mice were enriched in lesions compared to non-lesion white matter suggesting the immune environment promotes OPC recruitment and/or survival mechanisms. The reduction of GFP-negative differentiated oligodendrocytes (OL), representative of established OLs that existed prior to tamoxifen induction and survived the immune attack, was similar after adoptive transfer in both young and aged animals. A similar number of OLs was identified within lesions compared to non-lesion white matter also suggesting a relative preservation of OLs in lesions despite high inflammatory activity. Lineage traced GFP + OPCs differentiated within lesions and a similar degree of remyelination was found in both young and aged lesions. These findings indicate that young and aged OPCs respond similarly to a myelin-reactive T cell-mediated inflammatory attack and suggest that cell intrinsic pathways support OPC and OL survival in an inflammatory environment.

## Methods

### Animal care and use

Young (10–15-week-old) and aged (47–58 week-old) *Pdgfra*-*Cre*^*ERT*^ hemizygote +/− (Jackson Laboratory 018280); *RCE*− *Rosa26*^*flstopflEGFP*^ hemizygote +/− (Jackson Laboratory 032037-JAX, MMRRC stock #32027) female and male mice were assigned to experiments based on availability. Age indicated represents animal age at start of experiment when tamoxifen and immunization initiated. Mice were maintained on a 12-hour light/dark cycle, housed in cages of five or less, and provided food and water ad libitum. All animal experiments were conducted in accordance with the National Institute of Health Guide for the Care and Use of Laboratory Animals and were approved by the Animal Care and Use Committee at The Ohio State University (2023A00000086).

### Adoptive transfer/Experimental autoimmune encephalomyelitis (EAE)

Transgenic mice *Pdgfra*-*Cre*^*ERT*^; *RCE* were administered tamoxifen (Sigma T5648) intraperitoneally (100–150ul of 10mg/ml stock reconstituted in corn oil for working dose of 75mg/kg/day) daily for 5 days to induce recombination starting two weeks prior to adoptive transfer. Mice used for transmission electron microscopy (TEM) did not receive tamoxifen. T cells for adoptive transfer were generated from wildtype 8–10 week old (“young”) female C57BL/6J (Jackson Laboratory 000664). Wild-type mice were anesthetized with intraperitoneal injection of ketamine (90mg/kg)/xylazine (10mg/kg) and immunized with four subcutaneous flank injections with a total of 0.4mg of myelin oligodendrocyte glycoprotein (MOG)_35–55_ peptide (1mg/ml, biosynthesis) resuspended in Complete Freund’s Adjuvant (CFA) with concentration of 5mg/ml heat killed *M. tuberculosis*. After 10 days immunized wild-type mice were euthanized with CO_2_ and spleen and lymph nodes were dissected and placed on ice in EAE media: RPMI (ThermoFisher 21870084), 10% FBS (GeminiBio 100-106-500), P/S (Fisher 15-140-148), 1x GlutaMAX (Fisher 35-050-061), 1x NEAA (ThermoFisher 11140050), 1mM sodium pyruvate (Fisher 11-360-070), 55nM beta-mercaptoethanol (ThermoFisher 21985023). Spleens and lymph nodes were mashed across filter, washed with EAE media, and centrifuged at 800g 5min. Spleens were resuspended in RBC lysis buffer (ThermoFisher 00-4300-54) for 2 minutes at room temperature followed by quenching with EAE media, centrifuged at 800g 5min, and washed with EAE media. Spleen and lymph nodes were resuspended in EAE media with MOG_35–55_ peptide (50mg/ml, biosynthesis), recombinant mouse IL-23 (8ng/ml, R&D 1887-ML), recombinant mouse IL-1a (10ng/ml, Thermofisher 211–11A), anti-IFN-g (10mg/ml, Bio X Cell BE0055) at volume of 5×10^6^ cells/ml in T75 flasks. Cells suspensions were incubated at 37°C in 5% CO_2_ incubator for 4 days. After *ex*-*vivo* Th17 polarization cells were collected into conical tubes, centrifuged 800g 5min, filtered and washed twice with MACs buffer (0.5% BSA and 2mM EDTA in PBS). CD4 T cell selection was performed with mouse CD4 (L3T4) microbeads (Miltenyi Biotec 130-117-043) with LS column per Miltenyi protocol. Eluted CD4 + cells were washed in MACs buffer twice and 4×10^6^ cells were injected intraperitoneally into tamoxifen-administered transgenic mice. For naïve/non-adoptive transfer animals, littermates of adoptive transfer animals that received tamoxifen were separated into naïve cages. Mice were monitored daily starting on day 4 post-adoptive transfer with weights and EAE clinical scoring scale. EAE scoring scale: 0.5 reduced tail tone; 1 complete loss of tail tone; 1.5 slight gait imbalance; 2 gait imbalance with footfalls or spinning/ataxia; 2.5 hindlimb foot drag able to move above hip; 3 partial to complete hindlimb paralysis, not able to move either hindlimb above hip, moving around cage with forelimbs; 3.5 complete hindlimb paralysis, hindlimbs held to one size, moving around cage with forelimbs; 4 complete hindlimb paralysis, hindlimbs held to one size, no movement around cage but alert; 4.5 moribund not alert; 5 death. Mice were sacrificed at days 9–10 post-adoptive transfer with most severe mice sacrificed on day 9 time-point to avoid death and following end-point euthanasia protocols (euthanasia at score ≥ 4 within 24 hours or sustained weight loss ≥30% for 3 days). All mice in this study were between clinical scores of 2.5–4.5 at day of sacrifice and a total of 5 separate cohorts of adoptive transfer were performed. Clinical scores from mice used for immunohistochemistry and transmission electron microscopy were combined and there were no significant differences in clinical scores between mice that were administered tamoxifen for immunohistochemistry analysis and those that did not for TEM analysis. Several cohorts of adoptive transfer mice were used to optimize TEM fixation protocol, and clinical scores were combined for all adoptive transfer transgenic mice. Animals that were sacrificed or reached score 5 (death) were not included as data-points on subsequent days on EAE clinical scoring graphs.

### Immunohistochemistry

Mice were anesthetized with intraperitoneal injection of ketamine (90mg/kg)/xylazine (10mg/kg) and perfused transcardially with 15ml of 0.1M phosphate-buffered saline (PBS) followed by cold 4% paraformaldehyde (PFA) in PBS (ThermoScientific J19943.K2). Spinal cords were dissected out of the spinal column and post-fixed in 4% PFA/PBS at 4°C overnight then cryoprotected in 30% sucrose/1×PBS at 4°C for at least 2 days. Prior to embedding two transverse cuts were performed (at the end of the cervical enlargement and beginning of lumbar enlargement) to separate the spinal cord into cervical, thoracic and lumbar segments. Spinal cord segments were embedded in Tissue-Tek OCT compound (Sakura) with a cross-section of the beginning of each spinal cord segment facing the cutting edge of the block. Spinal cord blocks were sectioned at 15mm thickness with Leica CM1950 cryostat onto Superfrost Plus microscope slides (Fisher 12-550-15) with each 8–10 series of slides having four sets of cervical/thoracic/lumbar transverse sections (12 total sections) representing ~ 600mm distance from first to fourth section in each region to obtain representative analysis across each spinal cord segment on one slide. Slides were stored at −80°C until use. For immunostaining slides were permeabilized with 1×PBS + 0.1% Triton-X100 (PBST) for 10 minutes followed by outlining of sections with PAP pen (ThermoFisher 008899). To prevent non-specific binding, 5% normal goat serum (JacksonImmuno 005-000-121) in PBST was added to sections for 1 hour at room temperature followed by addition of primary antibodies in same blocking solution overnight at 4°C. Slides were washed in PBST three times 5 minutes each followed by incubation in secondary antibodies in blocking buffer for 1 hour at room temperature. Slides were washed with PBST twice for 5 minutes each, once with PBS for 5 minutes then mounted with ProlongGold antifade (ThermoFisher P36935). For FluoroMyelin co-stained slides, FluoroMyelin Red (Invitrogen F34652) was added at 1:300 dilution in PBS after last wash and incubated for 20 minutes followed by PBS wash and mounting. The following primary antibodies were used: chicken anti-GFP (Abcam ab13970, 1:1000), rabbit anti-Olig2 (MilliporeSigma ab9610, 1:1000), rabbit anti-Aspa (MilliporeSigma ABN1698, 1:250), rat anti-CD45 (MilliporeSigma 05–1416, 1:500), rabbit anti-degraded myelin basic protein dMBP (MilliporeSigma ab5864, 1:1000), rabbit anti-Pdgfra (CellSignaling 3174, 1:500) and mouse anti-neurofilament heavy NF-H (EnCor MCA-AH1). The following secondary antibodies were used all purchased from Invitrogen and used at dilution of 1:500: Alexa Fluor 488 goat anti-chicken IgY (H + L) (A-11039), Alexa Fluor 594 goat anti-rat IgG (H + L) (A-21247), Alexa Fluor 647 goat anti-rabbit IgG (H + L) (A-21245), Alexa Fluor 647 goat anti-mouse IgG (H + L) (A-21236).

### Immunohistochemistry imaging and analysis

Images of immunohistochemistry stained spinal cord sections were acquired using Olympus IX83 inverted microscope with Zeiss ZEN microscopy software. Tiled images of transverse spinal cord sections at a resolution of 0.65pixel/mm (10x objective) were obtained for GFP/Olig2, GFP/Aspa, and dMBP stains. Higher resolution tiled images at 0.325um/pixel (20x objective) were taken for GFP/Aspa/CD45, neurofilament and FluoroMyelin stains. For manual-counting, image stacks were analyzed in ImageJ/Fiji using channels tool, cell counter and ROI manager. For macro-analyzed images (DAPI/GFP/Olig2, DAPI/GFP/Aspa and DAPI/GFP/NF stains), images were exported as non-compressed TIFF grayscale stack of channels. Macros were developed using a combination of background subtraction, moments thresholding and analyzing particle ImageJ/Fiji functions to identify positive cells. In refinement of macros for individual stains, quality assessment images with overlay of counted objects were analyzed for accuracy and parameters were adjusted for each macro to obtain the highest accuracy of cell detection. After importing the spinal cord section image stack, the macros prompt the user to outline regions of interest (ROI)− whole cord, gray matter and lesions. Lesions were identified and outlined by the presence of DAPI hypercellularity which correlated with CD45 infiltrate. Non-lesion white matter counts were generated by subtracting whole cord counts from gray matter and total lesion counts, this was due to the ease in outlining gray matter during the macro execution compared to total non-lesion white matter. For Aspa macro counts total lesions within each spinal cord section were outlined as one lesion. In NF macro and DAPI/GFP/Aspa/CD45 manual counts individual lesions were outlined separately to increase power of analysis given potential variability across lesions. For neurofilament quantification, DAPI/GFP/NF 20x tiled images were analyzed with NF macro allowing users to outline lesions based on DAPI hypercellularity and NF positive axonal density was calculated for individual lesions. For dMBP quantification DAPI/GFP/dMBP 10x tiled image stacks were quantified in ImageJ/Fiji by outlining total white matter area and white matter with positive dMBP staining for each spinal cord section. For FluoroMyelin quantification, DAPI/GFP/Fluoromyelin 20x tiled image stacks were quantified by outlining total white matter and demyelinated white matter without Fluoromyelin.

To generate non-biased transverse spinal cord cross-section counts throughout the cervical, thoracic and lumbar spinal cord, all spinal cord sections without significant folds or other tissue processing artifacts were imaged. Adoptive transfer sections without lesions were not quantified in order to focus on spinal cord regions with inflammatory infiltrate. Sections quantified per animal: GFP/Olig2 hand counts 6–16, GFP/Aspa/CD45 hand counts 3–6, GFP/Aspa macro 6, GFP/NF macro 3–8, GFP/FlouroMyelin 6–9, GFP/dMBP 6–12. Sections counts were not averaged across individual animals to focus on comparisons on an individual section/region level which allowed for analysis of enrichment within lesions compared to non-lesion white matter. Lesion to non-lesion cell density ratios were generated by calculating the ratio of average lesional density/average non-lesional density for each spinal cord transverse section. All macro codes have been made publicly available in GitHub-https://github.com/coleayden/spinalcordmacros.

### Transmission electron microscopy (TEM)

Young (11-week-old) and aged (48–53-week-old) OPC lineage tracing mice (*Pdgfra*-*Cre*^*ERT*^; *RCE*-*Rosa26*^*flstopflEGFP*^) that did not receive tamoxifen in the same adoptive transfer cohorts (aged represented at age of experiment start when tamoxifen protocol/immunization was initiated) were used for transmission electron microscopy experiments to assess remyelination. On day 10 post-adoptive transfer young and aged EAE mice were anesthetized with intraperitoneal injection of ketamine (90mg/kg)/xylazine (10mg/kg) and perfused transcardially with 15ml of 0.1M PBS followed by cold 4% PFA (EMS 15714-S) and 5% glutaraldehyde (EMS 16210) in 80mM sodium cacodylate and 9mM calcium chloride dihydrate in EM grade dH_2_O (EMS 22800–01) pH 7.4. The spinal cord was dissected out of the column and fixed for 24 hours in perfusion buffer. Spinal cord was cut into 2mm transverse segments on spinal cord matrix (EMS 69085-C) and 3–5 lumbar and 3–5 cervical blocks per animal were washed with wash buffer (70mM sodium cacodylate, 70mM sodium chloride, 0.5mM calcium chloride, 1mM magnesium chloride hexahydrate in EM grade dH_2_O) for 30 minutes followed by post-fixation in 1% osmium tetroxide (EMS 19152) in 100mM sodium cacodylate for 1 hour protected from light. Blocks were washed with EM grade dH_2_O twice 2 minutes each then transferred to glass vials followed by dehydration (50% EtOH, 70% EtOH, 80% EtOH, 95% EtOH, 100% EtOH × 2, 100% propylene oxide 2 times, with each wash 30 minutes). Blocks were incubated overnight in 1:1 Embed 812 resin (EMS 14120, medium hardness concentration):propylene oxide (EMS 20412) at room temperature. Solution was changed to 3:1 Embed 812 resin:propylene oxide for 24 hours followed by 100% Embed 812 resin for 24 hours at room temperature. Embedding molds were filled with fresh Embed 812 resin and individual spinal cord blocks were transferred to molds and baked at 60°C for 24 hours. Semi-thin sections from lumbar spinal cord blocks were cut on Leica Ultracut UCT at 1mm thickness with Histo Diamond Knife (EMS 60-HIS) and stained with 1% toluidine blue to visualize lesions. Ultrathin sections for TEM were cut on Ultra Diamond Knife (EMS 40-UL) at 70nm thickness on Ultramicrotome Reichert Ultracut E and mounted onto grids (Pelco 200 mesh 1GC200). Grids were stained using Hiraoka staining kit (EMS 71560–00) with uranyl acetate alternative (EMS 22405) for 20 minutes followed by rinses in EMS grade dH_2_O 30 seconds 3 times, then incubation in 3mg/ml lead citrate in 0.1N NaOH in dH_2_O for 3 minutes. Grids were washed with 20mM NaOH followed by 2 washes with dH_2_O for 30 seconds then air dried. Grids were imaged on FEI Tecnai G2 Spirit TEM and ventral side was identified by the presence of ventral central vein and ventromedial white matter regions were imaged at 1150x magnification. All lumbar blocks were sectioned, imaged and quantified. All lumbar blocks from aged and young animals contained lesions with notable loss of myelin and presence of immune infiltrate in this region. The degree of immune infiltrate was classified as small or large based on the extent of immune cells and demyelination present within the 1150x lesion image with small lesions noted as lesions without notable demyelination but with sparse inflammatory cells. TEM lesion images acquired at 1150x resolution representing one grid box were analyzed in MyelTracer (Kaiser et al. 2021) with users selecting and manually outlining myelin sheaths if needed for all axons with ≥2μm given the difficulty in outlining smaller diameter axons at this magnification and desire to quantify remyelination which is more feasible for large diameter axons. Axon diameter and axon plus myelin diameters and calculated g-ratios were exported and compiled in excel and g-ratios were plotted with a cutoff of 2mm axon diameter in GraphPad Prism. The percentage of myelinated axons with g-ratio ≥ 0.8 was calculated for each 1150x lesion image.

### Flow cytometry

Transgenic *Pdgfra*-*Cre*^*ERT*^; *RCE− Rosa26*^*flstopflEGFP*^ mice that received tamoxifen and Th17 T cells or no Th17 T cells (naïve) as described above were sacrificed at 9–10 days post adoptive transfer for flow cytometry analysis of GFP reporter expression. Mice were anesthetized with intraperitoneal injection of ketamine (90mg/kg)/xylazine (10mg/kg) and perfused transcardially with 15ml of 0.1M PBS. Brains and spinal cord were dissected and dissociated in 20U/ml papain and 100U/ml of DNase in papain buffer (1mM EDTA, 5mM L-cystine, EBSS pH 7.4–7.6) for 30–45 minutes in 37°C 5% CO_2_ incubator with 3–4 manual trituration steps during incubation to dissociate into single cell suspension. Dissociated cells were spun at 800g 5min 4°C and resuspended in 30% Percoll (VWR 89428–524) in FACs buffer (2% FBS in PBS no cations) and spin 1000g 10 minutes 4°C speed 8. Myelin layer was removed and pellet was resuspended in FACs buffer followed by transfer over 70mm filter, PBS wash and resuspension in PBS with Fc block (BD Biosciences rat anti-mouse CD16/CD32 552142, 1:200) and viability stain (eBioscience 65-0865-14, 1:1000) for 10 minutes on ice. Cells were washed in FACs buffer and resuspended in primary antibody CD45 e450 (Invitrogen 48-0451-82 1:100) in FACs and incubated 30 minutes on ice followed by wash and resuspension in FACs. Flow cytometry of spinal cord and brain samples was performed with BD FACS Melody 4-way sorter and analyzed in FlowJo software.

### Statistics and reproducibility

No statistical methods were used to predetermine sample sizes without prior data of oligodendroglial counts in spinal cord adoptive transfer (AT). GraphPad Prism was used to generate graphs and perform statistical analysis. EAE scoring and quantification was blinded by use of genotyping animal ID that was later linked to a sample group in GraphPad prism analysis. A target sample size of 6 mice per group for immunohistochemistry and 4 mice per group for TEM were set for our analysis. For IHC analysis, average EAE score at sacrifice for young AT was 3.3 (minimum 2, maximum 4.5) and aged AT 3.7 (minimum 2.5, maximum 4.5) with male and females in both groups. A minimum of 4 animals per group and 3 sections per animal were analyzed for IHC outcomes and individual section and lesion data-points were represented to allow for more robust statistics given potential variability on a micro and macroenvironment level within different lesions and sections. For transmission electron microscopy a total of 4 young and 6 aged *Pdgfra*-*Cre*^*ERT*^; *RCE− Rosa26*^*flstopflEGFP*^ Th17 AT mice were sacrificed and processed with an average EAE score at sacrifice for young was 2.3 (minimum 2, maximum 2.5) and aged AT 3.6 (minimum 2.5, maximum 4) with males and females in both groups. All lumbar spinal cord blocks were quantified from these animals. In each figure and legend, the number of biological replicates and regions of interest (ROI) quantified, statistical test and significance levels are indicated. Datasets were considered significantly different at p < 0.05.

## Results

### Lineage tracing confirms that both young and aged OPCs persist in an inflammatory environment

**Figure 1:**
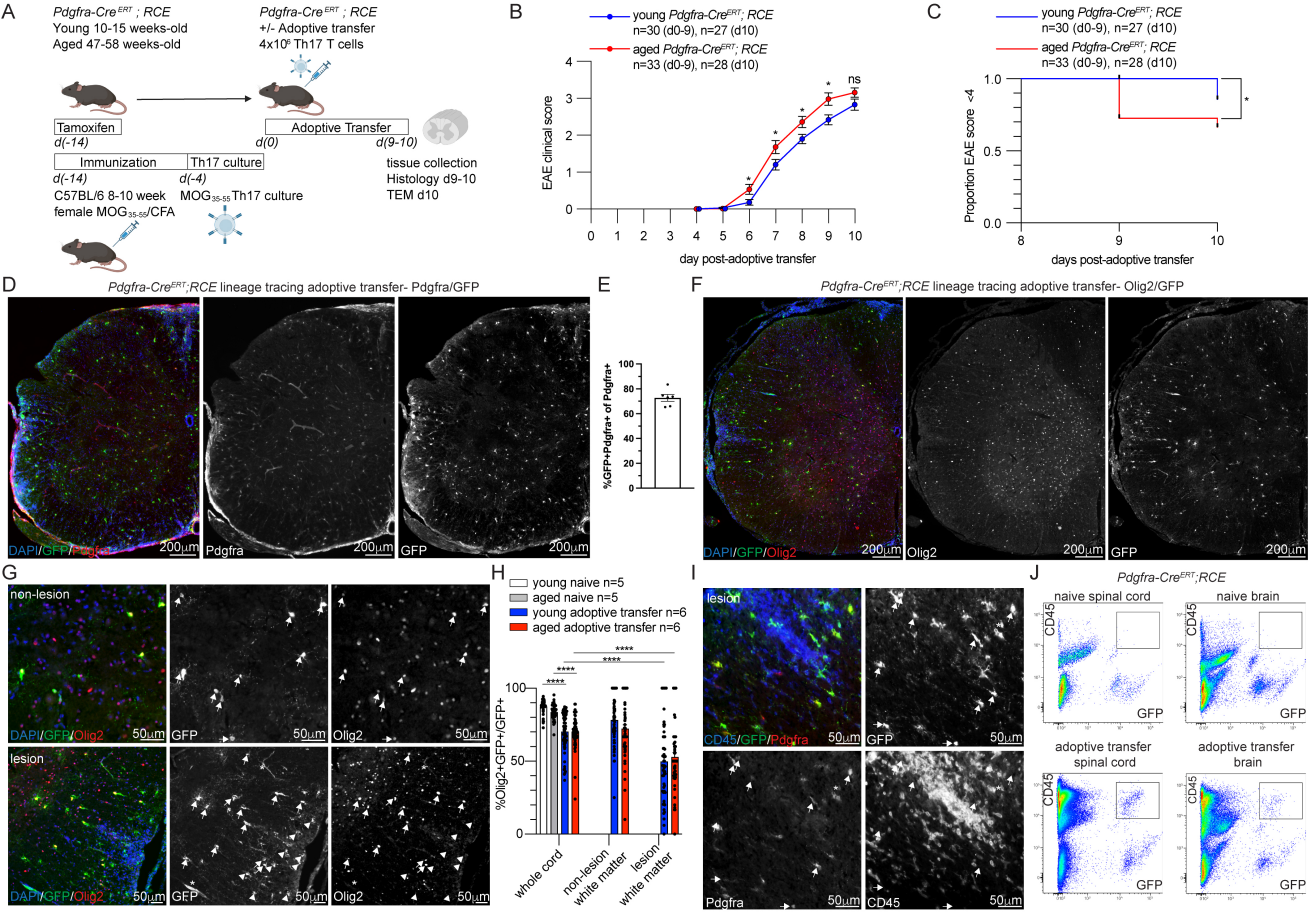
Lineage tracing strategy for labeling OPCs in young and aged adoptive transfer **A**. Experimental strategy for analysis of oligodendroglia using lineage tracing in young and aged *Pdgfra*-*Cre*^*ERT*^*;RCE* mice in naïve and Th17 adoptive transfer conditions. Created in BioRender. Harrington, E. (2026) https://BioRender.com/yutcpke. **B**. Aged *Pdgfra*-*Cre*^*ERT*^*;RCE* adoptive transfer animals have significantly higher EAE clinical scores at days 6–9 compared to young. Unpaired t-test, ns p>0.05, * p≤0.05. **C**. Kaplan-Meier survival analysis of proportion of animals with EAE score of less than 4 (≥4 quadriplegia, euthanasia endpoint) in young and aged adoptive transfer with significantly higher proportion of aged animals reaching score ≥4 by day 10. Logrank (Mantel-Cox) test, * p≤0.05. **D**. Representative 10x tiled images of *Pdgfra*-*Cre*^*ERT*^*;RCE* Th17 adoptive transfer spinal cord with lineage-traced OPCs labeled with GFP antibody and co-labeled with OPC marker Pdgfra. **E**. Quantification of OPC recombination efficiency in naïve young and aged *Pdgfra*-*Cre*^*ERT*^*;RCE* spinal cord with mean percentage of Pdgfra+ cells that are GFP+. n=6 animals. **F**. Representative 10x tiled images of *Pdgfra*-*Cre*^*ERT*^*;RCE* Th17 adoptive transfer spinal cord co-labeled with pan-oligodendroglial marker Olig2. **G**. Lineage traced GFP+ cells co-localize with Olig2 (arrows) in spinal cord non-lesion and lesion areas. Some GFP-positive cells are Olig2-negative (arrowheads) particularly within lesions. GFP+Olig2− cells with fibroblast morphology indicated with asterisks. **H**. Quantification of the percentage of lineage traced GFP-positive cells that express Olig2 in naïve, adoptive transfer, aged and young spinal cord regions. Average lesional density is represented for each spinal cord section. Welch’s t test, ****p<0.0001. **I**. Representative image of lineage-traced *Pdgfra*-*Cre*^*ERT*^*;RCE* adoptive transfer spinal cord lesion co-labeled with GFP/Pdgfra/CD45. GFP+Pdgfra+CD45− OPCs (arrows) and GFP+Pdgfra+CD45+ cells (asterisk) are present within lesions. **I**. Flow cytometry of lineage traced *Pdgfra*-*Cre*^*ERT*^*;RCE* naïve and adoptive transfer brain and spinal cord with GFP+CD45+ cells (box) in adoptive transfer but not naïve brain and spinal cord. n= animal number, error bars standard error of mean (SEM).

To identify differences in the response of young and aged OPCs to an adaptive auto-immune response, tamoxifen was administered to young (10–15-week-old) and aged (47–58 week-old) *Pdgfra*-*Cre*^*ERT*^+; *RCE*-*Rosa26*^*flstopflEGFP*^ mice that stably express GFP upon upregulation of *Pdgfra* in OPCs (Fig. 1A). Two weeks after tamoxifen induction, myelin oligodendrocyte glycoprotein (MOG)-reactive T cells were generated from young (8–10 week) female donors and polarized into Th17 phenotype ex-vivo followed by adoptive transfer into young and aged *Pdgfra*-*Cre*^*ERT*^; *RCE− Rosa2*^*flstopflEGFP*^ mice for lineage tracing of OPCs. Spinal cord was collected at 9–10 days post-adoptive transfer, corresponding to the peak disease state, for immunohistochemistry and transmission electron microscopy (TEM) based quantification of myelin sheath thickness. Naïve/non-adoptive transfer control animals received tamoxifen without adoptive transfer and were sacrificed in the same cohorts.

Aged adoptive transfer mice had significantly higher experimental autoimmune encephalomyelitis (EAE) clinical scores relative to young adoptive transfer mice on days 6–9 (Fig. 1B) with earlier onset to quadriplegia (Fig. 1C). Others have shown that the more severe clinical course in aged animals is not rescued by young bone marrow reconstitution indicating that host CNS environment was responsible for increased disease severity (37). In these prior studies, aged microglia demonstrated a more reactive pro-inflammatory transcriptional signature (37). As aged adoptive transfer animals have a short lifespan due to disease severity, this analysis focused on differences between young and aged OPCs at the onset of peak clinical disease severity days 9–10. As the maximal immune infiltrate in the Th17 adoptive transfer model is in the spinal cord (38, 39), histological analysis was performed on transverse sections of spinal cord to permit comparison of lesional oligodendroglia to surrounding non-lesional white matter. After 3.5 weeks after initiation of tamoxifen administration in *Pdgfra*-*Cre*^*ERT*^mice, GFP co-localized with the OPC marker Pdgfra (Fig. 1D). The *Pdgfra*-*Cre*^*ERT*^ line demonstrated high recombination efficiency with > 70% of Pdgfra + cells expressed GFP (Fig. 1E). Additionally, GFP co-localized with the pan-oligodendroglial marker Olig2 (Fig. 1F), however an Olig2−/GFP + population was consistently identified, particularly within lesions (Fig. 1G–H). Olig2−/GFP + non-oligodendroglial lineage cells expressed Pdgfra and CD45 (Fig. 1I) which was confirmed by flow cytometry of adoptive transfer brain and spinal cord (Fig. 1J) suggesting that Olig2−/GFP + population represented host hematopoietic cells that migrated into lesions. Given the presence of GFP + non-oligodendroglial cells within lesions in this *Pdgfra*-*Cre*^*ERT*^ line, subsequent quantitative analysis of OPC behavior was performed using immunohistochemistry for the OPC lineage restricted markers (Olig2 and Aspa).

### Young and aged lineage traced OPCs are enriched within lesions

**Figure 2:**
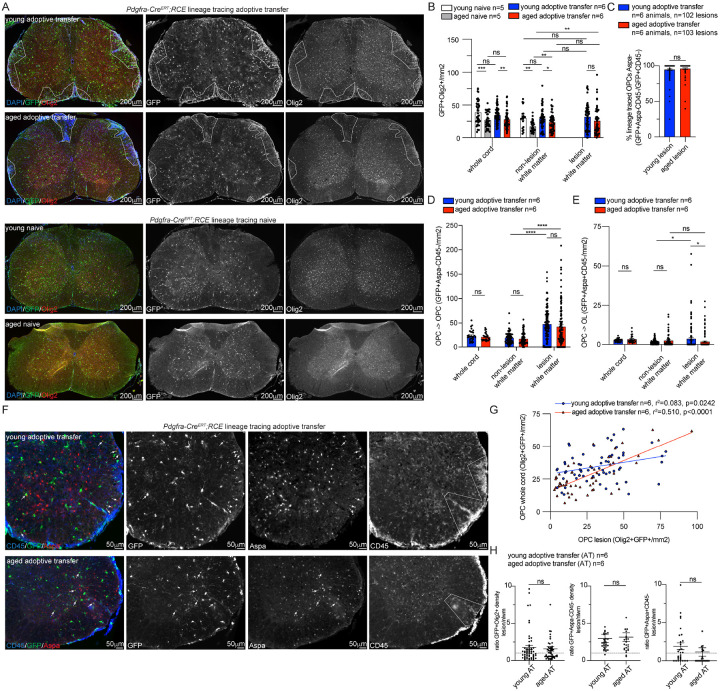
Young and aged lineage traced OPCs are enriched within adoptive transfer lesions **A**. Representative images of young and aged lineage traced OPC (*Pdgfra*-*Cre*^*ERT*^*;RCE*) naïve and adoptive transfer (AT) spinal cord sections stained for pan-oligodendroglial marker (Olig2), lineage traced OPCs (GFP) and DAPI (lesions outlined). **B**. Quantification of lineage traced OPC (Olig2+GFP+) densities. Average lesional density is represented for each spinal cord section. **C**. Quantification of percentage of lineage traced OPCs (GFP+CD45−) that remain OPCs (GFP+Aspa-CD45-) within young and aged AT lesions within individual lesions. **D**. Quantification of density of lineage traced OPCs that remained OPCs (GFP+Aspa-CD45−) with individual lesions, non-lesion white matter and whole cord. **E**. Quantification of density of lineage traced OPCs that differentiated into OLs (GFP+Aspa+CD45-) with individual lesions, non-lesion white matter and whole cord. **F**. Representative images of young and aged *Pdgfra*-*Cre*^*ERT*^*;RCE* AT spinal cord stained for GFP, Aspa, and CD45 (lesion area outlined). OPCs that remain OPCs (GFP+Aspa-) and OPCs that have differentiated (GFP+Aspa+, arrows) are present. **G**. Simple linear regression analysis of Olig2+GFP+ whole cord density compared to average total lesion density on an individual spinal cord section level. **H**. Quantification of the ratio of oligodendroglial density in lesions (average lesional density) compared to non-lesion white matter (nlwm) on an individual spinal cord section level. Ratio greater than 1 (dotted line) indicates enrichment of OPCs in lesions compared to nlwm. n= animal number, error bars standard error of mean (SEM), group comparisons Welch’s t test, p values: ns p>0.05, * p≤0.05, **p≤0.01, ***p≤0.001, ****p<0.0001.

To characterize the effect of aging on OPC behavior in naïve versus adoptive transfer animals, lineage tracing of OPCs was performed by double staining for GFP to identify populations that expressed Pdgfra at the time of tamoxifen induction and Olig2, a pan oligodendroglial marker (Fig. 2A). The density of lineage traced OPCs (GFP + Olig2+) in whole spinal cord sections, lesion white matter, and non-lesion white matter was quantified (Fig. 2B). The density of GFP + Olig2 + OPCs in both whole spinal cord and white matter regions was significantly lower in aged naïve spinal cord compared to young naïve spinal cord (Fig. 2B). However, after adoptive transfer, the density of GFP + Olig2 + OPCs in aged animals was significantly increased in both lesional and non-lesional white matter compared to aged naïve animals (Fig. 2B). These data indicate that despite the lower baseline density of OPCs in naïve aged animals, aged OPCs can still proliferate and mount a compensatory response in the face of an inflammatory insult. These findings suggest that OPCs are relatively preserved within lesions irrespective of age and aged OPCs retain the capacity to proliferate in response to the inflammatory insult.

To determine whether GFP + OPCs differentiated into mature oligodendrocytes or remained OPCs, co-labeling for GFP, the mature oligodendrocyte marker (Aspa) and the pan- hematopoietic marker (CD45) was performed (Fig. 2C–F). Consistent with the methodology applied to quantify OPCs density previously, the density of each individual lesion was quantified and represented as a separate data point (Fig. 2B). Regardless of host age, > 95% of GFP + cells in spinal cord lesions were GFP+/Aspa−/CD45− suggesting that the majority of Pdgfra + OPCs in lesions do not transition into mature oligodendrocytes after a demyelinating inflammatory insult (Fig. 2C). Despite the low rate of differentiation into mature oligodendrocytes, both young and aged adoptive transfer animals had a significantly higher density of OPCs (GFP + Aspa-CD45−) in lesions compared to non-lesion white matter indicative of a proliferative and/or migratory response (Fig. 2D). After adoptive transfer, the density of GFP + Aspa-CD45− OPCs was not significantly different between age groups in any region (Fig. 2D). Although newly formed oligodendrocytes (GFP + Aspa + CD45−) (Fig. 2E,F) were present throughout the spinal cord in both young and aged adoptive transfer animals, these differentiated OPCs were a rare finding (Fig. 2F). While a significantly higher density of newly formed oligodendrocytes was found in lesions relative to non-lesion white matter of young animals, there was no significant difference in the density of newly formed oligodendrocytes in lesion versus non-lesion white matter of aged animals (Fig. 2E). This indicates that while aged OPCs mount a proliferative response, they have a lower rate of differentiating into myelinating oligodendrocytes. To determine if the density of GFP + Olig2 + OPCs in lesions was correlated with density in the surrounding white matter in the same spinal cord section, we performed a linear regression analysis (Fig. 2G). After adoptive transfer in aged and young animals, a positive correlation was identified between the OPC density in lesions and OPC density in the remainder of the transverse section of the spinal cord white matter (Fig. 2G). Given the significant correlation between whole cord OPC density and lesion OPC density indicative of a global OPC response, the ratio of newly formed oligodendrocyte density in lesion versus non-lesion regions for each spinal cord cross section was calculated (Fig. 2H). A ratio greater than one indicates a relative enrichment of cells within lesions compared to non-lesion white matter. GFP + Olig2 + oligodendrocyte lineage cells and GFP + Aspa-CD45 OPCs were enriched in lesions compared to non-lesion white matter in both young and aged lesions. GFP + OPCs that differentiated into newly formed mature oligodendrocytes (GFP + Aspa + CD45−) were enriched in young lesions but not in aged lesions. Thus, while both young and aged OPCs respond to inflammation via proliferation, migration and/or upregulation of cell intrinsic survival mechanisms triggered by the inflammatory lesion microenvironment, young OPCs appear to have a superior capacity to generate myelinating oligodendrocytes.

### Adoptive transfer reduces the density of mature oligodendrocytes in the spinal cord

**Figure 3:**
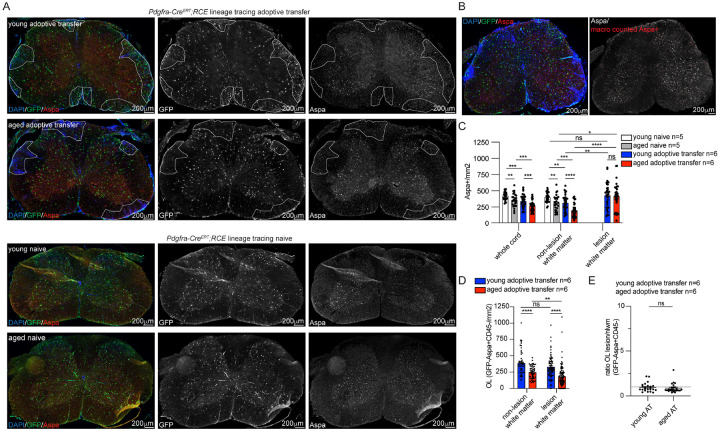
Differentiated oligodendrocytes are reduced in adoptive transfer **A**. Representative images of spinal cord sections from young and aged lineage traced OPC line (*Pdgfra*-*Cre*^*ERT*^*;RCE*) in adoptive transfer and naïve conditions stained for differentiated oligodendrocyte marker (Aspa), lineage traced OPCs (GFP) and DAPI hypercellularity indicating lesion areas (outlines). **B**. Representative adoptive transfer spinal cord section stained for DAPI/GFP/Aspa (left panel) and processed with GFP/Aspa macro with macro counted cells outlined in red overlayed on grayscale of raw image (right panel). **C**. Macro quantification of Aspa+ OL densities in naïve and adoptive transfer spinal cord. Average lesional density is represented for each spinal cord section. **D**. Manual quantification of GFP− OLs (GFP-Aspa+CD45-) in adoptive transfer non-lesion and lesion white matter with a significant decrease in OLs in aged lesions compared to both young lesions and aged non-lesion white matter. **E**. Quantification of the ratio of OL density (GFP-Aspa+CD45-) in lesions (average lesional density) compared to non-lesion white matter (nlwm) on an individual spinal cord section level. OLs are present at similar densities in lesion compared to nlwm at an individual sectional level. Dotted line = 1. n= animal number, error bars standard error of mean (SEM), group comparisons Welch’s t test, p values: ns p>0.05, * p≤0.05, **p≤0.01, ***p≤0.001, ****p<0.0001.

To determine the impact of an acute inflammatory demyelinating insult on established mature differentiated oligodendrocytes (OLs), Aspa + mature OLs were quantified in the spinal cord of naïve and adoptive transfer animals (Fig. 3A). Given the high density of Aspa + cells within the spinal cord ImageJ macros were developed for semi-automated cell counting of Aspa-positive cell densities in whole cord, non-lesion white matter, and lesion areas. The ImageJ macro accurately detected Aspa + cells throughout the spinal cord (Fig. 3B). The density of Aspa + OLs in both the whole spinal cord and white matter was significantly lower in aged naïve spinal cord animals compared to young naïve (Fig. 3C). After adoptive transfer, Aspa + OL densities were significantly reduced compared to naïve spinal cord in both age groups. Similar to findings for OPCs, the density of Aspa + OL in both whole cord and non-lesion white matter was significantly lower in aged compared to young adoptive transfer animals although this finding may be secondary to the overall loss of Aspa + OL observed with normal aging of naïve animals. In average lesion counts across sections, Aspa + OL density in lesions was not significantly different between age groups after adoptive transfer (Fig. 3C) and the OL density was enriched in lesion compared to non-lesion white matter. To quantify the impact of adoptive transfer on the density of established oligodendrocytes, GFP− OLs (GFP−/Aspa+/CD45−) were manually counted in lesion and non-lesion white matter (Fig. 3D). In aged adoptive transfer animals, the density of Aspa + OLs was significantly reduced in lesions compared to non-lesion white matter, but this reduction was not observed for lesions in young animals. These data indicate that either aged OLs are more vulnerable to an immune insult or simply reflect the more severe disease course observed in aged animals. To quantify whether established GFP−/Aspa + OLs were selectively reduced within lesions compared to non-lesion white matter, the ratio of mean GFP-Aspa + CD45− cell density in lesions compared to non-lesion white matter in the same spinal cord section was calculated for each transverse section (Fig. 3E). While a reduced density of established GFP−/Aspa + CD45− OLs was identified in aged compared to young animals after adoptive transfer, OLs were present at similar densities to normal appearing white matter in both young and aged animals. This indicates that established oligodendrocytes may have compensatory survival mechanisms that are preserved during aging and ensure survival of individual OLs in the face of an auto-immune response that targets myelin proteins.

### Aging potentiates axonal loss and myelin breakdown after adoptive transfer

**Figure 4:**
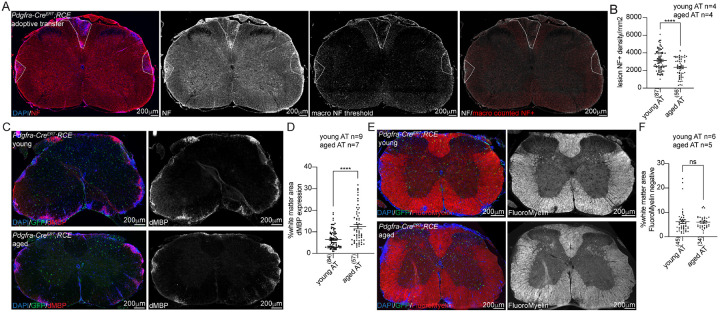
Axonal loss, myelin debris and demyelination in young and aged adoptive transfer **A**. Representative 20x tiled images of axonal density quantification by neurofilament-H (NF) in *Pdgfra*-*Cre*^*ERT*^*;RCE* adoptive transfer spinal cord section stained for DAPI/NF (left panel), NF only grayscale image (NF, middle left panel), NF macro thresholded image (macro NF threshold, middle right panel) and NF macro processed image with NF-positive axons outlined in red (right panel). Lesions outlined. **B**. NF macro quantification in young and aged adoptive transfer (AT) lesions with significantly less axonal density in aged lesions. **C**. Representative image of degraded myelin (dMBP) staining and GFP staining in young and aged adoptive transfer *Pdgfra*-*Cre*^*ERT*^*;RCE* spinal cord sections. **D**. Quantification of the percentage of white matter occupied by dMBP-positive staining in young and aged adoptive transfer spinal cord with significant increase in myelin debris in aged lesions. **E**. Representative images of FluoroMyelin and GFP staining in young and aged adoptive transfer *Pdgfra*-*Cre*^*ERT*^*;RCE* spinal cord sections. **F**. Quantification of the percentage of demyelinated white matter with absent FluoroMyelin staining in adoptive transfer spinal cord with no significant difference between young and aged. n= animal number, sections quantified indicated by number in parenthesis, error bars standard error of mean (SEM), group comparisons Welch’s t test, p values: ns p>0.05, ****p<0.0001.

To determine the effect of aging on the degree of axonal loss, myelin debris and demyelination after adoptive transfer, transverse spinal cord sections from young and aged adoptive transfer mice were stained for neurofilament (NF), degraded myelin basic protein (dMBP) and FluoroMyelin (total myelin) (Fig. 4). An ImageJ macro was developed to quantify axonal density in outlined regions of interest by automated counting of neurofilament-positive axons (Fig. 4A). Aged animals had a significantly higher degree of axonal loss within lesions compared to young animals (Fig. 4B). Similarly, a greater proportion of spinal cord white matter in aged mice contained degraded myelin compared to young mice (Fig. 4C–D), consistent with reports that aged macrophages are less efficient at clearing myelin debris (20). Although aged mice had an increase in myelin debris, there were no significant differences between age groups in the degree of demyelination quantified by the absence of FluoroMyelin staining (Fig. 4E–F).

### Remyelination is present to a similar extent in young and aged lesions

**Figure 5:**
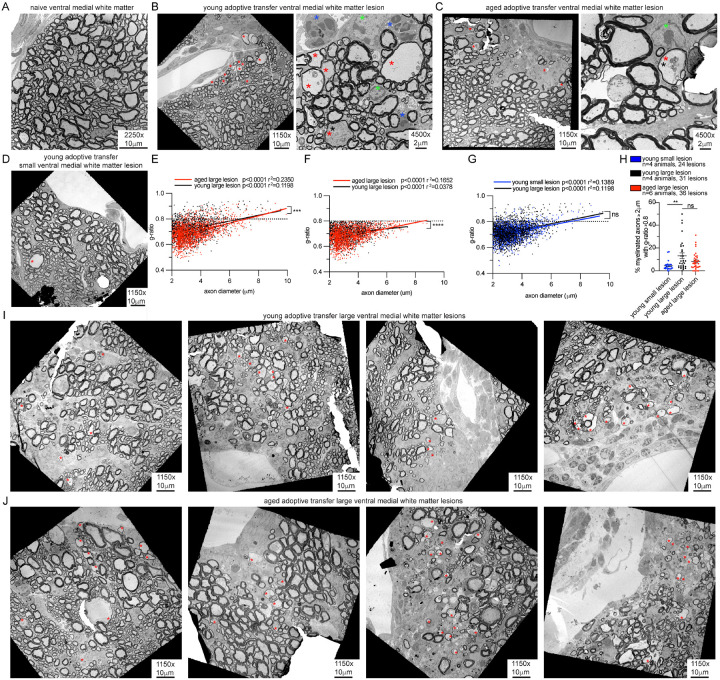
Remyelination in young and aged adoptive transfer lesions by transmission electron microscopy **A**. Transmission electron microscopy (TEM) of ventral medial spinal cord white matter of naïve/non-adoptive transfer young *Pdgfra*-*Cre*^*ERT*^*;RCE*. **B**. Representative images of young adoptive transfer ventral medial spinal cord lesion and **C**. Aged adoptive transfer ventral medial white matter lesion. Higher magnification image regions represented by white box on lower magnification images. Remyelinated axons with thin myelin sheath indicated with red asterisk. Infiltrating immune cells with polymorphic nuclei indicated with blue asterisks. Cells with condensed nuclear chromatin consistent with oligodendroglia indicated with green asterisks. Axons with increased electron dense material or mitochondria indicated with black asterisks. **D**. Representative images of young small adoptive transfer lesions with minimal immune infiltrate. **E**. Quantification of g-ratios in young and aged adoptive transfer large lesions. Axons with g-ratio ≥0.8 above dotted line represent thin myelin sheaths and remyelinated axons. Simple linear regression analysis. **F**. Linear regression analysis of g-ratios in young and aged adoptive transfer large lesions with removal of g-ratio datapoints ≥0.8 indicating significant difference in lineage regression lines driven by lower r-gatios in aged animals. **G**. Quantification of g-ratios in small compared to large young adoptive transfer lesions with simple linear regression analysis. **H**. Quantification of percent of myelinated axons with g-ratio ≥0.8 in individual lesions in young and aged adoptive transfer. Welch’s t-test. **I**. Representative images of large lesions from young adoptive transfer lesions. **J**. Representative images of large aged adoptive transfer lesions. Remyelinated axons with thin myelin sheath indicated with red asterisk. n= animal number, error bars standard error of mean (SEM), p values: ns p>0.05, **p≤0.01, ***p≤0.001, ****p<0.0001.

The relative preservation of OPCs and differentiated oligodendrocytes within lesions suggested that despite a robust inflammatory response, OPCs and OLs can maintain their numbers within lesions and OPCs can differentiate into mature OLs (Fig. 2E–F). To determine if oligodendrocytes can remyelinate in young and aged lesions, transmission electron microscopy (TEM) was performed on young and aged ventromedial white matter lesions (Fig. 5). This location was chosen based on the high rate of lesions, ability to identify landmarks with the presence of a ventral vein, and the consistent presence of large diameter axons and higher g-ratios (Fig. 5A) (40) that would facilitate the ability to detect thinner remyelinated axons. Toxin-mediated demyelinating models have demonstrated that remyelinated axons can be identified by thinner myelin sheaths (41–43). Perivascular and parenchymal immune infiltrates were identified in all ventral white matter lesions as well as high concentration of oligodendrocytes (Fig. 5B,C). Thin myelin sheaths consistent with remyelinated axons were present both in young (Fig. 5B) and aged lesions (Fig. 5C). Some remyelinated axons had electron dense cytoplasm and notable mitochondria (Fig. 5B,C), a potentially reversible early sign of ultrastructural axonal damage in the setting of inflammatory demyelination (44). Smaller lesions with sparse immune cells and no notable regions of demyelination were also present in young but not aged animals (Fig. 5D). Quantification of g-ratios was performed on 1150x lesion images to allow for quantification of remyelinated axons across a lesion. The g-ratio of myelinated axons with a diameter ≥2μm were quantified with MyelTracer (45). Axons with thin myelin sheaths were noted to have g-ratios of 0.8 or higher. Remyelinated axons with g-ratio ≥ 0.8 were found in both young and aged large lesions (Fig. 5E). G-ratio slopes were significantly different between large young and aged lesions (Fig. 5E), however this effect was also present after removal of axons with g-ratio ≥ 0.8 (Fig. 2F) confirming that the difference in slope was due to lower g-ratios and thicker myelin sheaths in aged animals. This finding is consistent with previous studies of human (46) and mouse spinal cord tracts (47). In young animals, there were no significant differences in G-ratios in large compared to small lesions (Fig. 5G). To determine whether the degree of remyelination was affected by aging, the percentage of myelinated axons with a g-ratio of ≥ 0.8 was calculated for each 1150x lesion image. The most extensive remyelination was found in large lesions with 10–15% of axons ≥2μm exhibiting a g-ratio of ≥ 0.8 (Fig. 5H). There was no significant difference between the percentage of remyelinated axons in young and aged large lesions. Small lesions in young animals had significantly less axons with thin myelin sheaths than large lesions. Representative images of large young and aged adoptive transfer lesions are shown (Fig. 5I,J). Despite more extensive axonal loss, increased myelin debris (Fig. 4C,D), and smaller numbers newly formed OLs (Fig. 2E) observed in aged lesions, the extent of remyelination is similar between age groups, even in the presence of a robust inflammatory infiltrate. Although the high clinical severity of aged animals precluded analysis of chronic time-points, both young and aged animals demonstrated the capacity to remyelinate at the early stage of the disease.

## Discussion

OPCs are dynamic cells with the ability to self-renew, migrate, and differentiate into mature, myelinating oligodendrocytes. Although the response of OPCs to demyelination has been investigated in using toxin-mediated models and genetic ablation strategies (5), the fate of OPCs, after exposure to a robust auto-immune inflammatory insult remains poorly understood. Biological aging influences multiple sclerosis disease activity and progression (13) and the response of OPCs in an aged environment is particularly relevant for a chronic disease that affects individuals at all stages of life. Aged OPCs retain the capacity to proliferate; (16) however, physiological aging is associated with a decreased rate of OPC proliferation (14, 16) and differentiation into mature OLs (14). Aged OPCs express higher levels of the cell cycle inhibitors p16 and p21, which may reflect age-associated senescence (48). In response to toxin-mediated demyelination, the rates of proliferation, recruitment, and differentiation in aged OPCs exhibit slowed kinetics; however, with time remyelination of aged animals catches up to young OPCs (17–19). One proposed mechanism for the slower kinetics is increased expression of factors that inhibit differentiation including HIF-1a and WNT (16).

To determine whether aging affects the response of OPCs to a strong adaptive immune response akin to an acute MS attack, we performed lineage tracing of OPCs in young adult and aged mice after adoptive transfer of young myelin reactive Th17 polarized T cells. Despite a lower baseline density of OPCs, aged OPCs respond to an acute inflammatory demyelination event leading to an increase in OPC density in both lesions and the surrounding spinal cord. In both aged and young animals, compensatory mechanisms result in an increase in OPC density in lesions compared to non-lesion white matter. In both young and aged animals, a small number of OPCs respond to an inflammatory demyelinating insult by differentiating into newly formed oligodendrocytes, although the response is less robust in aged animals. A similar extent of remyelination is observed in the spinal cord of aged animals despite increased myelin debris and increased axonal loss in aged lesions. During an acute demyelination event, mature oligodendrocytes are reduced both in young and aged spinal cord to a similar extent. We are unable to determine with TEM analysis alone if remyelination occurs from newly generated oligodendrocytes or existing differentiated oligodendrocytes, both of which can occur in toxin-mediated models (4). While OPCs differentiate to a lesser extent in aged adoptive transfer lesions (Fig. 2E), OPCs are enriched in both young and aged lesions (Fig. 2D, H). Further studies combining OPC lineage tracing with genetic reporter strategies (49) or immunogold labeling of lineage traced OPCs combined with TEM may offer insight into the origin of remyelinating oligodendroglia in immune-mediated models.

Previous studies combining genetic strategies to induce CNS interferon gamma production with toxin-mediated demyelination indicate that OPCs through expression of MHC class I may target themselves for destruction by CD8 T cells (32). We have found in this adoptive transfer model using myelin-reactive Th17 T cells that the presence of an inflammatory infiltrate provokes an increase in the density of OPCs in lesions followed by differentiation and remyelination in both young and aged animals. Our findings indicate that a robust inflammatory demyelinating insult stimulates recruitment and retention of OPCs to lesions regardless of age suggesting that the lesional microenvironment stimulates OPC responses. OPCs may have cell intrinsic compensatory strategies that allow them to survive and persist in the presence of T cells.

In the absence of an autoimmune attack, physiological aging of the CNS is associated with accumulation of CD8 T cells (50) and increased Stat1 interferon-response signatures in OPCs (16, 47). Aged oligodendroglia may be primed and more responsive to interferon gamma signaling through Stat1. In viral CNS infection models, oligodendrocytes that survive the acute viral infection upregulate MHC class I (51) and programmed death-ligand 1 (PD-L1) (52) and oligodendroglial PD-L1 expression may function to inhibit CD8 T cells activity (52). Stat1 and PD-L1 are also expressed by oligodendroglia in active EAE models (24, 49) and Stat1 may induce PD-L1 expression (53). Upregulation of interferon-response pathway in young and aged OPCs may in fact be beneficial for oligodendroglial survival allowing OPCs and OLs to evade the immune attack and dampen immune signaling. Further studies are needed to investigate the role of disease-associated oligodendroglia in inflammatory models to determine whether manipulation of immunoregulatory pathways in oligodendroglia promotes survival and remyelination.

## Conclusions

This study demonstrates that oligodendroglia in young and aged animals respond similarly to an acute auto-immune mediated demyelinating attack mediated by MOG-reactive Th17 T cells. Both aged and young OPCs are enriched in spinal cord lesions and a similar degree of remyelination is present in aged and young lesions. Lineage traced young and aged OPCs both demonstrated the ability to differentiate during auto-immune mediated demyelination. The ability of aged OPCs to respond similarly to young OPCs in the presence of an autoimmune attack suggests that remyelination and repair strategies that target OPCs may be promising strategy for people of all ages with MS.

## Data Availability

Macro codes have been made publicly available as described in methods. The datasets generated during the current study are available from the corresponding author on reasonable request.

## Abbreviations

AT: Adoptive transfer
Aspa: Aspartoacylase
CD45: Cluster of differentiation 45
CNS: Central nervous system
CFA: Complete Freund’s Adjuvant
dMBP: Degraded myelin basic protein
EAE: Experimental autoimmune encephalomyelitis
GFP: Green fluorescent protein
HIF-1a: Hypoxia inducible factor-1 alpha
IFN-g: Interferon gamma
MHC: Major histocompatibility complex
MOG: Myelin oligodendrocyte glycoprotein
MS: Multiple sclerosis
NF: Neurofilament
Nlwm: Non-lesion white matter
OPC: Oligodendrocyte progenitor cell
OL: Oligodendrocyte
Olig2: Oligodendrocyte transcription factor 2
Pdgfra: Platelet-derived growth factor receptor alpha
PD-L1: Programmed death-ligand 1
RCE: Rosa26 CAG-boosted EGFP reporter allele (*Rosa26*^*flstopflEGFP*^)
ROI: Regions of interest
SC: Spinal cord
TEM: transmission electron microscopy
Th17: T helper 17 cell
WT: Wild-type
